# Ex vivo dendritic cell-based (DC) vaccine pulsed with a low dose of liposomal antigen and CpG-ODN improved PD-1 blockade immunotherapy

**DOI:** 10.1038/s41598-021-94250-0

**Published:** 2021-07-19

**Authors:** Mona Yazdani, Zahra Gholizadeh, Amin Reza Nikpoor, Nema Mohamadian Roshan, Mahmoud Reza Jaafari, Ali Badiee

**Affiliations:** 1 Nanotechnology Research Center, Pharmaceutical Technology Institute, Mashhad University of Medical Sciences, Mashhad, Iran; 2 Department of Molecular and Comparative Pathobiology, Johns Hopkins University School of Medicine, Baltimore, MD 21205 USA; 3 Molecular Medicine Research Center, Hormozgan Health Institute, Hormozgan University of Medical Sciences, Bandar Abbas, Iran; 4 Immunogenetic and Cell Culture Department, Immunology Research Center, School of Medicine, Mashhad University of Medical Sciences, Mashhad, Iran; 5 Department of Pathology, School of Medicine, Mashhad University of Medical Sciences, Mashhad, Iran; 6 Biotechnology Research Center, Pharmaceutical Technology Institute, Mashhad University of Medical Sciences, Mashhad, Iran; 7 Department of Pharmaceutical Nanotechnology, School of Pharmacy, Mashhad University of Medical Sciences, P.O. Box 91775-1365, Mashhad, Iran

**Keywords:** Cancer microenvironment, Cancer therapy, Tumour immunology, Drug delivery, Nanotechnology in cancer, Oncology, Adjuvants, Cell vaccines, Melanoma, Cytokines, Immunotherapy

## Abstract

Lack of pre-existing tumor infiltrated T cells resulting in resistance to programmed cell death protein 1 (PD-1) blockade therapies can be solved by combining with anti-cancer vaccines and CpG-ODN in increasing T cell expansion and infiltration. Therefore, we prepared an ex vivo dendritic cell-based (DC) vaccine pulsed with a low dose of either liposomal or non-liposomal gp100 antigen (2.8 µg) plus CpG-ODN (800 ng) formulations and evaluated its anti-tumor activity in combination with anti-PD-1 therapy. Our results showed a combination of liposomal peptide plus CpG-ODN pulsed DC with anti-PD-1 antibody was more efficacious, as evidenced by a significant increase in T_eff_/T_reg_ TILs with a marked fourfold elevation of IFN-γ expression level in the tumor site of treated mice which reversed resistance to PD-1 blockade in a CD8 T cell-dependent manner. Furthermore, this combination also led to a remarkable tumor remission and prolonged survival rate in melanoma-bearing mice compared to non-liposomal peptide plus CpG-ODN or single-treated liposomal peptide formulations. Our results provide essential insights to devise combining regimens to improve the efficacy of immune checkpoint blockers even by a low dose of peptide and CpG-ODN.

## Introduction

Blockade of an inhibitory checkpoint pathway such as programmed cell death protein 1 (PD-1) has significantly improved the current landscape of cancer immunotherapy^1^. Despite inducing potent and durable anti-tumor immunity, this approach has a limited function as monotherapy due to the lack of pre-existing T cell infiltration into the tumor microenvironment (TME), which leads to resistance to PD-1 blockade therapy^2,3^. Poor immunogenicity of tumor-associated antigens (TAAs) and immunosuppressive characteristic of TME may cause primary resistance to PD-1 therapy^4^. There is emerging data pointing to the potential application of anti-cancer vaccines combined with monoclonal antibodies (mAb), resolving this obstacle^5,6^.

Successful anti-cancer immunotherapy is mainly dependent upon the presence and activation of antigen-specific cytotoxic T cells (CTLs) as essential arms in anti-cancer immunity^7^ and IFN-γ production needed for amplifying T cell responses^1^. Indeed, many TAAs as peptide vaccine inducing CTL responses have been extensively researched^8^, but most of them were self-antigens that faced significant barriers as immune tolerance^9^, besides their poor immunogenicity. The weak immune responses to TAAs may be due to their physical size, which are typically below the desired size for efficient uptake by dendritic cells (DCs)^10^. In most cases, peptides are routinely administered with an immune adjuvant^11^. In vivo administration of immunoadjuvant with glycoprotein 100 (gp100), as a melanoma TAAs, effectively increased the efficacy of vaccination against selected TAA^12^.

Co-delivery of TAAs with immune-inducing adjuvant like CpG-ODN to the same DCs efficiently enhance the uptake by DCs, accelerates the induction of the immune system, and extends the duration of the induced immune response^13^. In addition, co-delivery with immune adjuvant enables to reduce the dose of antigen^14^. In this regard, several delivery vehicles, such as liposomes, biodegradable particles, etc., were examined to optimize the antigen plus adjuvant delivery to the same DCs^15,16^. Beyond the standard properties, the synergic effect of CpG-ODN in combination with checkpoint blockers (i.e., PD-1 or CTLA-4), revived T cell activity, and improved survival rate identified it as an ideal candidate supporting immune checkpoint therapy (ICT) in preclinical cancer models^17,18^.

We evaluated the role of liposomal peptide delivery to dendritic cells and their use as DC vaccine in our previous study in which the significant efficacy of liposomal peptide pulsed DC vaccination in the enhancement of PD-1 blockade therapy was proved^19^. However, in this study, we investigated the role of CpG-OGN co-delivered with liposomal or non-liposomal peptide to ex vivo generated DCs as an anti-cancer vaccine in combination with PD-1 blockade therapy. We found mice that received liposomal peptide plus CpG-ODN showed a robust immune response and significantly prolonged survival rate compared to non-liposomal peptide plus CpG-ODN or single treated liposomal peptide formulations.

## Results

### Characterization of CpG-ODN formulations

As was shown in Table 1, the particle size of liposomal gp100 was 255.7 ± 5.43 nm. The addition of CpG-ODN to Lip-peptide led to a slight increase in the particle size resulting in particles with an average size of 266.4 ± 7.94 nm. It is noteworthy that the mixture of the peptide with CpG-ODN formed a particle with a size of 149.5 ± 35.11 nm and showed an almost narrow size distribution which is ideal for uptake by dendritic cells.

**Table 1:** DLS analysis. Characterization of peptide and cationic liposome formulations. All data of vesicle size, Polydispersity, and zeta potential represent Mean ± SD (N=3). Polydispersity, and zeta potential represent Mean ± SD (N=3).

Groups	Formulations	Particle size (nm)	PDI	Zeta potential (mV)
Peptide + CpG-ODN	–	149.5 ± 35.11	0.29 ± 0.11	− 6.42
Lip-peptide	DOTAP: Chol: GP100	255.7 ± 5.43	0.36 ± 0.05	7.10 ± 6.19
Lip-peptide + CpG-ODN	DOTAP: Chol: GP100: CpG-ODN	266.4 ± 7.94	0.41 ± 0.04	− 11.4 ± 5.24

### The frequency of T cells subpopulations in spleen and tumor site

**Figure 1:**
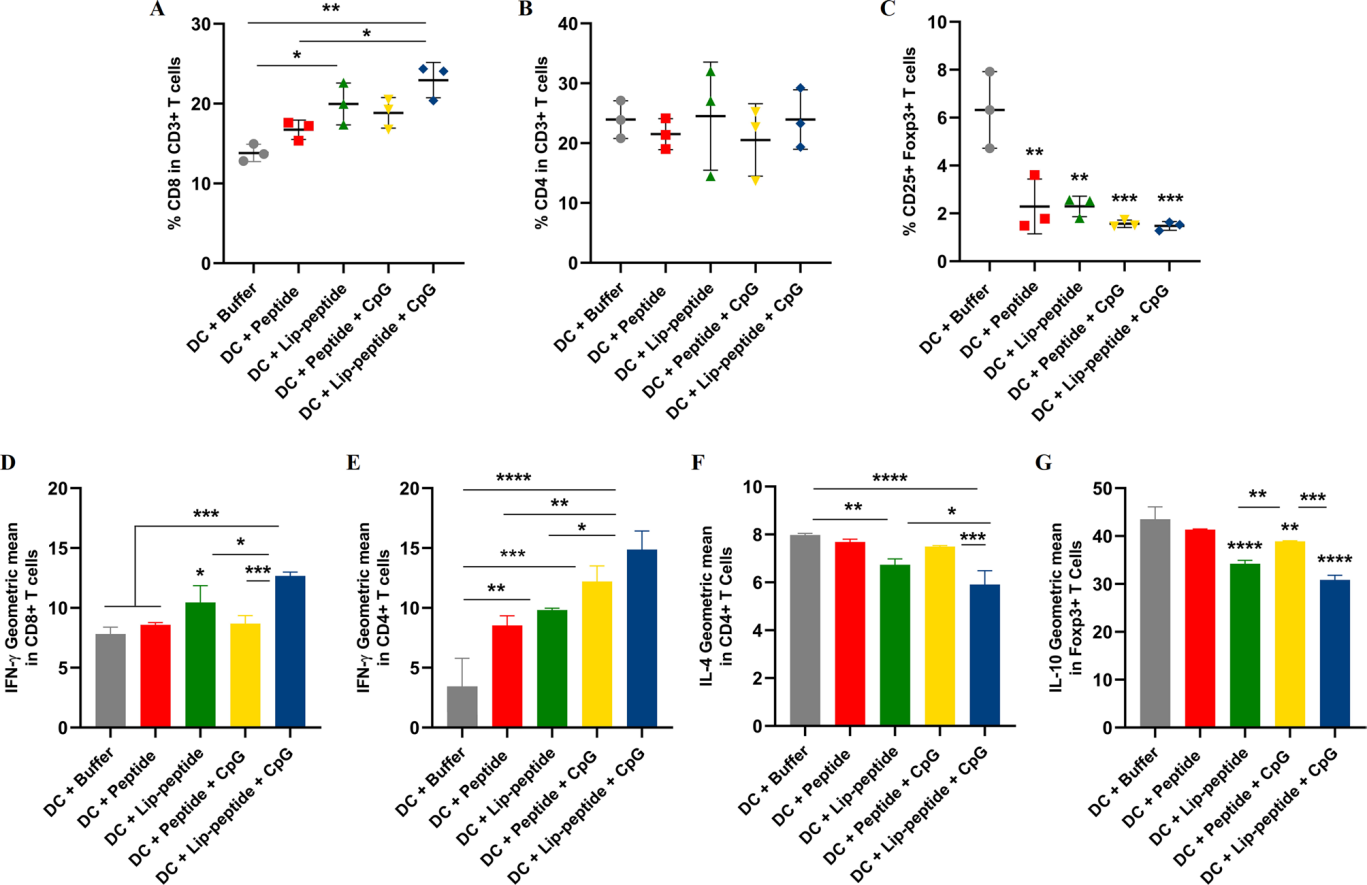
Immune cell subsets expansion and cytokine production within the spleen. The frequency of CD8+ T cells (**A**), CD4+ T cells (**B**), CD4+ Foxp3+ T cells (**C**) and median fluorescence intensity (MFI) of IFN-γ (**D**, **E**), IL-4 (**F**), IL-10 (**G**) cytokine profile of splenocytes of mice received the combination therapy. The data are presented as mean ± SD (N = 3). Statistically significant differences are shown as follows: *P < 0.05, **P < 0.01, ***P < 0.001, ****P < 0.0001.

Based on gating strategies (Fig. S1), combination therapy with liposomal peptide plus CpG-ODN led to a significant increase in the percentage of CD3+ CD8+ T cells compared to other groups (Fig. 1A), while no change was observed in the percentage of CD3+ CD4+ T cells between groups (Fig. 1B). All groups showed a significant decrease in the percentage of CD4+ CD25+ Foxp3+T cells followed combination therapy than the control group (Fig. 1C). In splenocytes, an increased level in IFN-γ + production by CD8+ T cells was shown in liposomal peptide plus CpG-ODN over other groups (Fig. 1D). Similarly, the IFN-γ expression by CD4+ T cells was greatly increased in the liposomal peptide plus CpG-ODN group compared to single-treated or control groups (Fig. 1E). IL-4 production by CD4+ T cells was also significantly decreased in the liposomal peptide plus CpG-ODN group (Fig. 1F). A significant decrease in the number of CD4+ CD25+ Foxp3+ T cells in the spleen notably attenuated IL-10 production in both liposomal peptide plus CpG-ODN and liposomal groups (Fig. 1G). The results of the percentage of cytokine-producing T cells in the spleen were in line with the results of cytokine production (MFI) (Fig. S2A).

**Figure 2:**
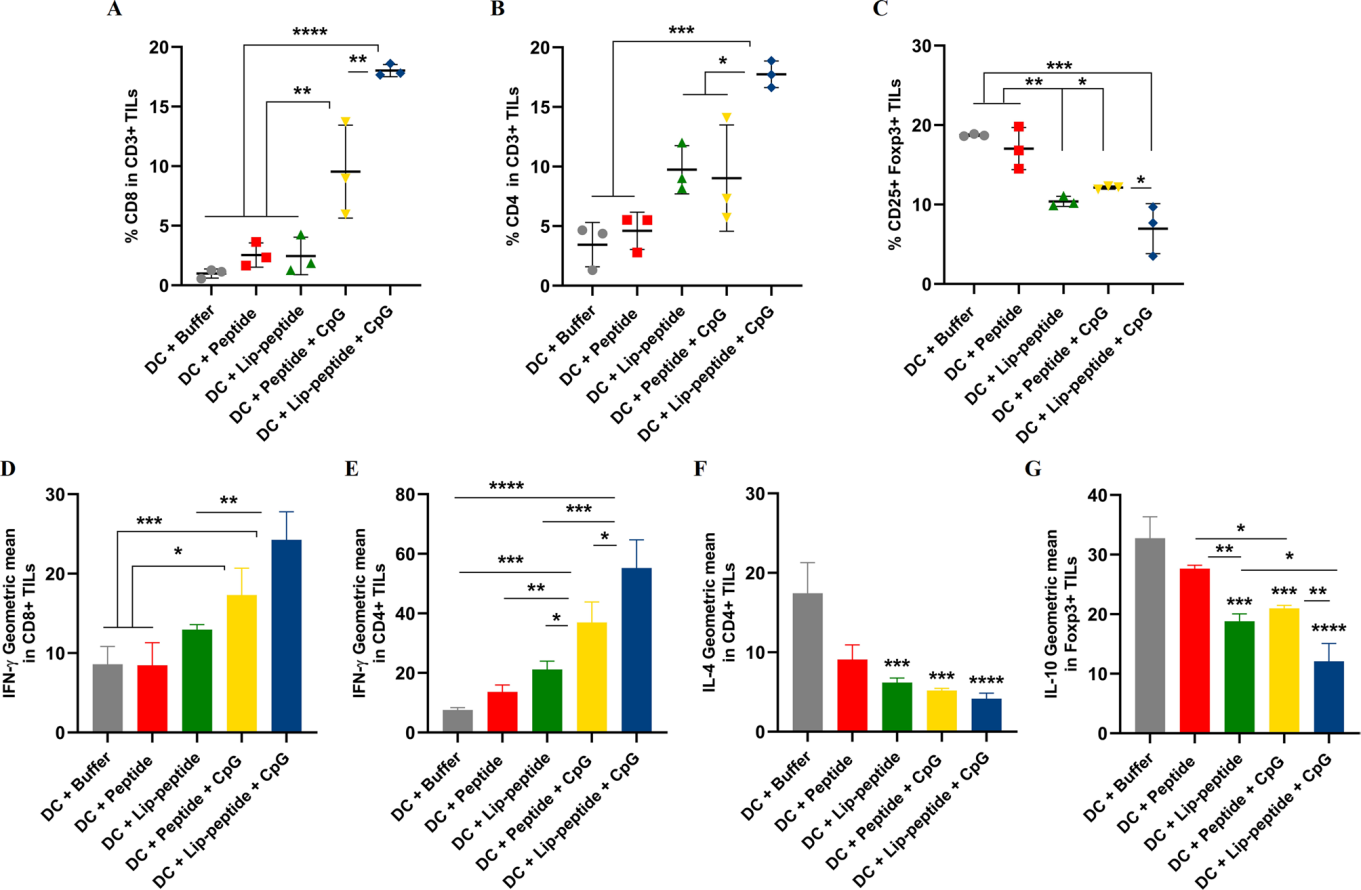
Immune cell subsets infiltration and cytokine production within the tumor site. The frequency of CD8+ TILs (**A**), CD4+ TILs (**B**), CD4+ Foxp3+ TILs (**C**) and median fluorescence intensity (MFI) of IFN-γ (**D**, **E**), IL-4 (**F**), IL-10 (**G**) cytokine profile of tumor cells of mice received the combination therapy. The data are presented as mean ± SD (N = 3). Statistically significant differences are shown as follows: *P < 0.05, **P < 0.01, ***P < 0.001, ****P < 0.0001.

In the tumor site, a significant difference in the proportion of CD3+ CD8+ TILs were shown over peptide plus CpG-ODN (*P* < 0.01) and single treated groups (*P* < 0.0001) (Fig. 2A). Unlike the results in the spleen, in the tumor site, a significant increase was seen in the percentage of CD3+ CD4+ TILs (Fig. 2B) compared to other groups. A significant decrease in the number of CD4+ CD25+ Foxp3+ TILs in the tumor site was observed in the liposomal peptide plus CpG-ODN treated group (Fig. 2C). Similarly, the IFN-γ production by CD8+ TILs were greatly increased in liposomal peptide plus CpG-ODN group compared to single-treated or control groups. In the peptide plus CpG-ODN treated group, the frequencies of CD8+ TILs were significantly increased (*P* < 0.01), with an increase observed in IFN-γ expressing TILs (*P* < 0.05). However, there was no significant difference over the liposomal group (Fig. 2D). In IFN-γ production by CD4+ TILs this difference in liposomal peptide plus CpG-ODN group was also shown over peptide plus CpG-ODN and liposomal groups (*P* < 0.05 and *P* < 0.001, respectively). The same difference was detected over single-treated groups (Fig. 2E). IL-4 production by CD4+ TILs was significantly decreased in liposomal peptide plus CpG-ODN group and also in liposomal or non-liposomal peptide plus CpG-ODN groups compared to control group (Fig. 2F). IL-10 production in liposomal plus CpG-ODN group was lower than peptide plus CpG-ODN and liposomal groups (*P* < 0.01 and *P* < 0.05 respectively) in tumor site (Fig. 2G). Like spleen, in tumor site also the results of the percentage of cytokine-producing TILs confirmed the MFI results (Fig. S2B).

**Figure 3:**
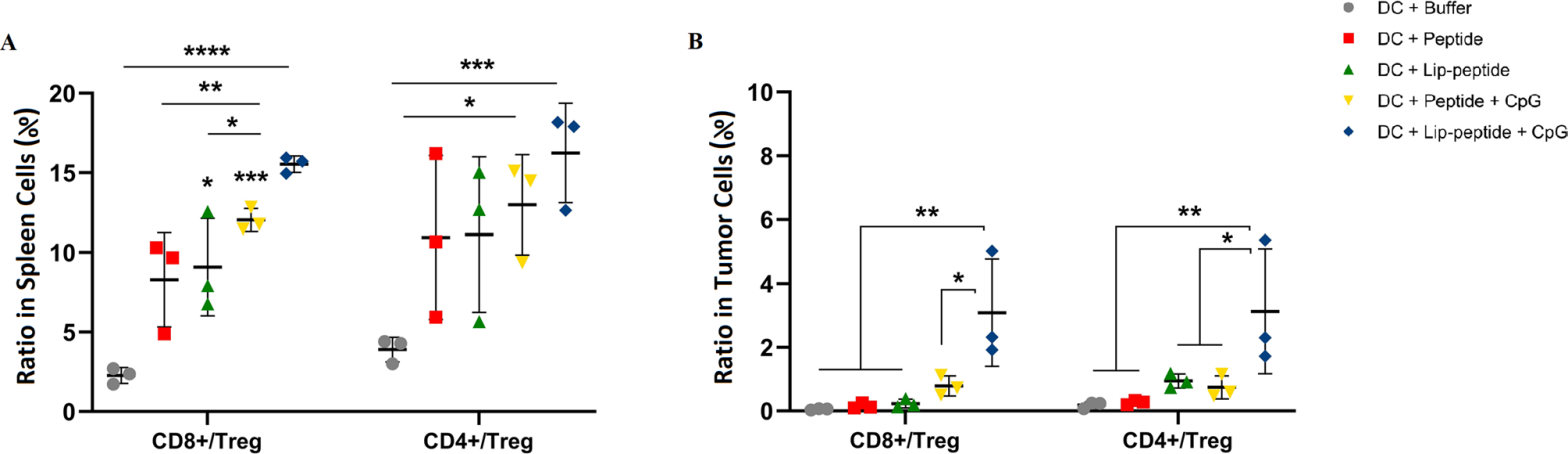
The relative changes in effector/regulatory T lymphocytes ratio in spleen and tumor site. (**A**) Represents the ratio of CD8 and CD4/Treg T cells and (**B**) represents the ratio of CD8 and CD4/Treg TILs. The data are presented as mean ± SD (N = 3). Statistically significant differences are shown as follows: *P < 0.05, **P < 0.01, ***P < 0.001, ****P < 0.0001.

The CD8 T effector to Treg cell ratio was most significant in the liposomal gp100 plus CpG-ODN group compared to single-treated or control groups in both sites. In the non-liposomal gp100 plus CpG-ODN group, the difference was observed just over the control group in the spleen (Fig. 3A,B). In CD4 to Treg cell ratio, the difference was observed in liposomal gp100 plus CpG-ODN group in both sites (Fig. 3A,B). The presented results suggested that infiltration and expansion of T cells had occurred within the tumor site.

### Cytotoxic activity of splenocytes against B16F10 cells

**Figure 4:**
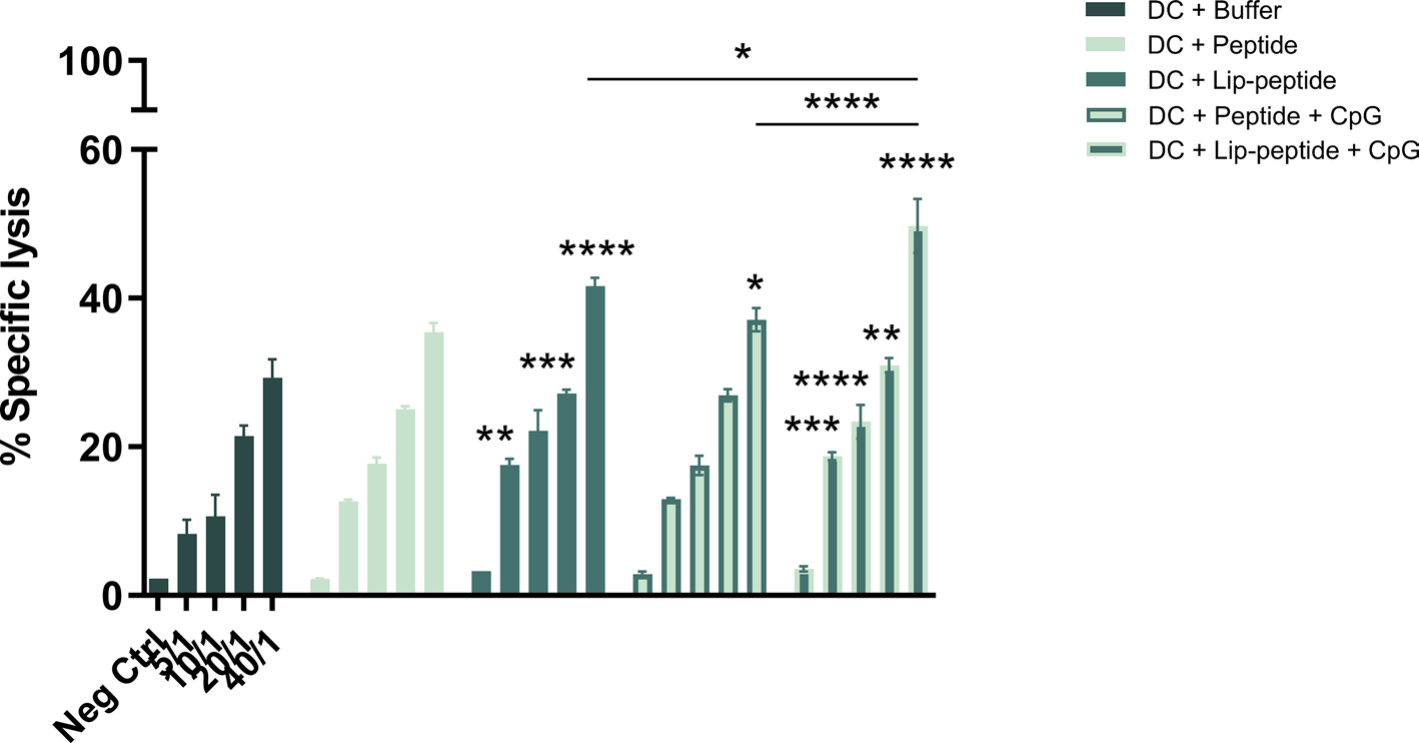
Antigen-specific cytotoxic cytolysis. Splenocytes isolated from mice received combination therapy. Specific lysis (%) of Calcein AM labeled B16F10 cells expressing gp100 and CT26 (gp100 negative) in co-cultured with splenocytes is depicted per E: T (E: effector cells and T: target cells) ratio. The data are presented as mean ± SD (N = 3). Statistically significant differences are shown as follows: *P < 0.05, **P < 0.01, ***P < 0.001, ****P < 0.0001.

Analysis of gp100-specific CD8+ T cells revealed the superior induction of CD8+ T cells when CpG-ODN was co-delivered with antigen. Splenocytes collected from mice immunized with liposomal peptide plus CpG-ODN exhibited the highest cytotoxicity when co-cultured with B16F10 murine melanoma cells in all ratios compared to the control group and also in 40/1 ratio over peptide plus CpG-ODN and liposomal peptide treated groups. The significant difference in other groups compared to the control group was indicated on each ratio (Fig. 4).

### Immune responses measured by ELISPOT assay

**Figure 5:**
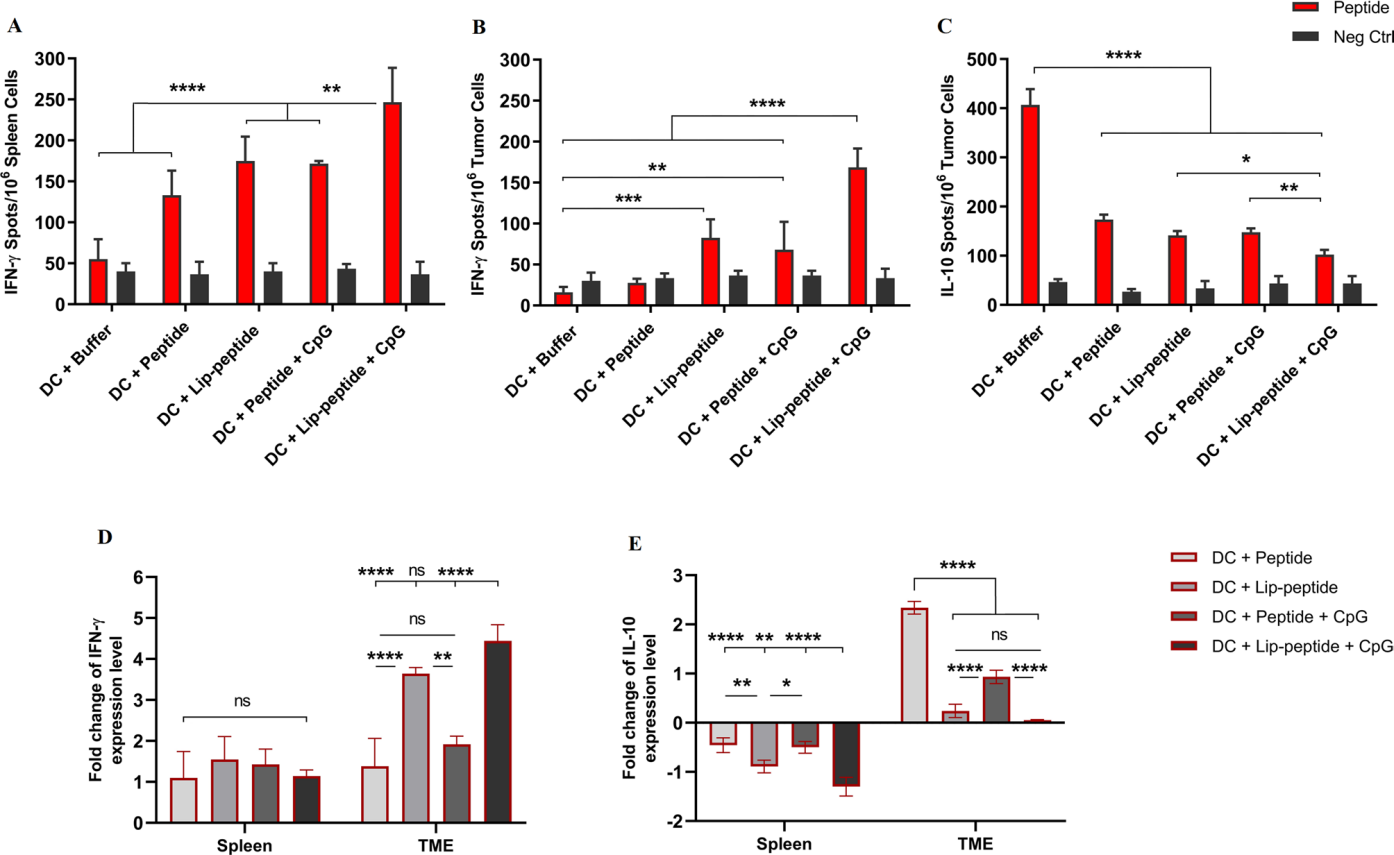
Analysis of cytokine-secreting cells by ELISPOT and cytokine mRNA expression analysis by real-time PCR. IFN-γ secreting cells in the spleen (**A**) and tumor site (**B**) and IL-10 secreting cells in tumor site (**C**) of mice received combination therapy. The mRNA expression levels of two selected cytokines—IFN-γ (**D**) and IL-10 (**E**)—in spleen and tumor site followed combination therapy. The relative mRNA expression levels of these genes were normalized relative to housekeeping gene, GAPDH. The data are presented as mean ± SD (N = 3). Statistically significant differences are shown as follows: NS *P* > 0.05, *P < 0.05, **P < 0.01, ***P < 0.001, ****P < 0.0001.

CpG-ODN dramatically enhanced liposomal and non-liposomal peptide-induced immune responses. We found that the IFN-γ-secretion was significantly increased when the liposomal peptide was combined with CpG-ODN and delivered to DC. This has led to the highest number of IFN-γ secreting cells in both spleen and tumor sites. Results showed a significant difference in liposomal peptide plus CpG-ODN compared to the peptide plus CpG-ODN and liposomal peptide groups, which in tumor site was higher (*P* < 0.0001) than spleen site (*P* < 0.01). The data also indicated that the co-delivery of peptide and CpG-ODN resulted in significantly increased numbers of IFN-γ secreting TILs compared to the control group (Fig. 5A,B). In evaluating IL-10 secreting Treg cells, both CpG-ODN containing groups had decreased the number of cells than the control group (*P* < 0.0001) significantly. Also, between these two groups, the liposomal peptide plus CpG-ODN group had a significantly lower number of cells than the non-liposomal peptide plus CpG-ODN group (*P* < 0.01) (Fig. 5C).

### mRNA expression level in spleen and tumor site

The level of IFN-γ expression showed no significant difference in the spleen, but in tumor biopsies, in the liposomal peptide plus CpG-ODN group, the significant difference was at the highest level compared to peptide groups (*P* < 0.0001). However, there was no significant difference over the liposomal peptide group in the tumor. IFN-γ gene expression analysis also revealed that the gene was enriched in tumor biopsies from mice treated with liposomal formulation than peptide and non-liposomal plus CpG-ODN groups. Analysis of IFN-γ in the non-liposomal peptide plus CpG-ODN group was not significantly different from the peptide group (Fig. 5D). IL-10 expression correlated positively with the activity of regulatory T cells. Our results showed a significantly lowest IL-10 expression in the liposomal peptide plus CpG-ODN group than other groups in the spleen. In tumor biopsies, all groups showed a significantly lower expression level of the IL-10 peptide group (*P* < 0.0001). Despite the result of the IFN-γ gene, non-liposomal peptide plus CpG-ODN treatment led to decreased IL-10 expression level compared to the peptide group (Fig. 5E).

### Enhanced infiltration of T cells into the tumor site

**Figure 6:**
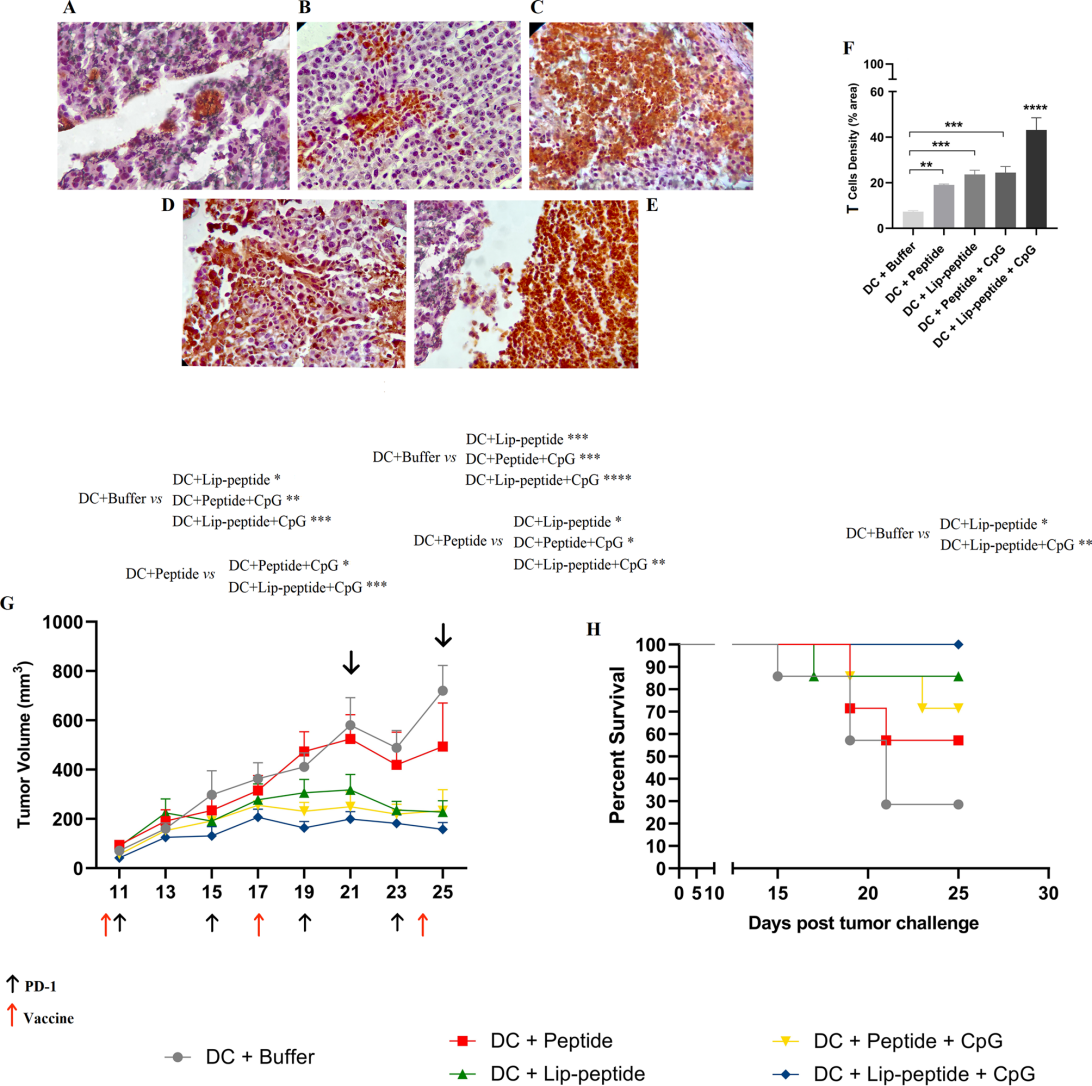
T cell infiltration and Therapeutic efficacy of different ex vivo DC-based vaccination groups in combination with PD-1 blockade in B16F10 melanoma model. Representative IHC results of tumor sections with different intensities of PD-1 staining. T cells, either clustered or dispersed, are stained dark brown. Images of tumor section from (**A**) DC + Buffer, (**B**) DC + Peptide, (**C**) DC + Lip-peptide, (**D**) DC + Peptide + CpG-ODN and (**E**) DC + Lip-peptide + CpG-ODN, Scale bars, 50 µm. (**F**) T-cell densities (mean ± SD, N = 3) as percentage of anti-PD-1-stained area against the total area. (**G**) Tumor growth curves of treated mice with tumor volume at any specific time point during treatment for a 25-day study period. This graph is also illustrating the timeline of vaccination and PD-1 therapy. *P* values correspond to tumor volume on days 21 and 25. Data are presented as mean ± SEM, N = 7. (**H**) Kaplan–Meier survival curve during 25 days therapeutic immunotherapy. The Log-rank test used for the significant difference between all combination groups (N = 7). *P* values correspond to survival rate on day 25. Statistically significant differences are shown as follows: *P < 0.05, **P < 0.01, ***P < 0.001, ****P < 0.0001.

IHC analysis of biopsies of the treated groups, in comparing to control group (Fig. 6A), all the groups had significantly increased the number of TILs even peptide (Fig. 6B) or liposomal (Fig. 6C) single treated groups. Of note, infiltration of T cells in peptide plus CpG-ODN group (Fig. 6D) was higher in tumor biopsies than peptide group (Fig. 6B), suggesting the requirement of co-delivery of peptide and CpG-ODN adjuvant in inducing a potent T cell infiltration followed by PD-1 blockade therapy. The significant large clusters of PD-1+ TILs were co-localized in tumor site of liposomal peptide plus CpG-ODN group followed by PD-1 blockade therapy which was significantly different over all other groups (Fig. 6E,F).

### Co-delivery of CpG-ODN enhanced anti-tumor immunity

To investigate the importance of co-delivery of CpG-ODN and tumor antigen to DC as an anti-cancer vaccine, we used B16F10 tumor-bearing mice received combination therapy with PD-1 blockade. Indeed, pulsing DC with liposomal peptide plus CpG-ODN showed a more potent anti-tumor effect with significant tumor regression as shown in Fig. 6G and Fig. S3, which took about 40 days to reach the maximum volumes of tumors (> 1000 mm^3^; Table 2). On day 21, 4 days after the second vaccination, the tumor volume in mice treated with liposomal peptide plus CpG-ODN pulsed DCs was the lowest compared to peptide pulsed and control groups. On day 25, the tumor volume in the peptide group was a bit decreased that led to changes in significant difference with liposomal peptide plus CpG-ODN group, unlikely; the difference was significantly increased compared to control group (Fig. 6G), which subsequently resulted in 100% surviving mice (Fig. 6H). Our results also showed a significant difference between this group and those received the liposomal peptide (37 days, *P* < 0.05; Table 2). In addition, all animals in the non-liposomal peptide plus CpG-ODN group had a slower tumor progression (Fig. 6G) which exhibited a significant delay to reach the endpoint (38 days) than the peptide group (25 days, *P* < 0.01; Table 2) and 70% of the animals were survived (Fig. 6H).

**Table 2:** Therapeutic efficacy data of different DC-based vaccine formulations in B16F10 tumor mice model.

Treatment groups	TTE^a^ (days ± SD)	TGD^b^ (%)	MST^c^ (days)	ILS^d^ (%)
DC + Buffer	23.66 ± 5.45	0	21	0
DC + Peptide	25.07 ± 9.02	5.94	> 25	> 13.64
DC + Lip-peptide	37.19 ± 10.47^e,f^	57.15	> 25	> 13.64
DC + Peptide + CpG-ODN	38.56 ± 12.36^e,f^	62.94	> 25	> 13.64
DC + Lip-peptide + CpG-ODN	40.29 ± 8.64^e,f,g^	70.25	> 25	> 13.64

Data are shown as mean ± standard deviation (N = 7).

^a^Time to reach the endpoint.

^b^Tumor growth delay.

^c^Median survival time.

^d^Increased life span.

^e,f^Indicates a significant difference compared with DC + Buffer and DC + Peptide groups, respectively (*P* < 0.01).

^g^Indicates a significant difference compared with the DC + Lip-peptide group (*P* < 0.05).

## Discussion

Here, we show that ex vivo co-delivery of CpG-ODN with antigen is required to generate an effective DC vaccine that synergizes with checkpoint blockers to induce long-lasting anti-tumor immunity in melanoma. Biologic therapies incorporating PD-1 blocking antibodies in immunogenic cancers like melanoma have resulted in the extended survival rate of patients in randomized controlled trials^20,21^, which were dependent mainly on a pre-existing infiltrating population of cytotoxic T cells^22^. Despite the good benefit of anti-PD-1 therapy, the induced resistance during treatment has limited its efficacy^4^. The effective anti-tumor immune response induced by synthetic CpG-ODN agonists of TLR9 in preclinic and clinical studies with anti-PD-1 antibody therapy revealed the critical role IFN-γ signaling for anti-PD-1 response^23^. This information allowed us to design combinatorial strategies changing the tumor microenvironment to successfully overcome the resistance towards PD-1 blockade in the mouse melanoma model.

The remarkable difference in the efficacy of combination therapy is usually correlated with the co-delivery of CpG-ODN with antigen to the same DC, which is a prerequisite for antigen presentation and subsequent priming of antigen-specific T cells by DCs^24^. Moreover, local depot formation at the injection site also improves DCs uptake^25^. Depending on the physicochemical property of the antigen molecule, co-administration of TLRs with antigen will result in particle formation because of electrostatic interaction, with a desirable size for uptake by DCs^11^.

Our study suggests that besides additive immunostimulatory properties of CpG-ODN, our peptide and CpG-ODN have simultaneously produced a nanoparticle complex which increased its immunogenicity effect significantly. As was shown, both the number of T cells and circulating IFN-γ levels in the peptide-pulsed group were significantly weaker than the peptide-CpG-ODN complex pulsed group. We speculated that a significantly higher accumulation has occurred by peptide-CpG-ODN particles in a competition between the peptide-CpG-ODN nanoparticle and free peptide size. The particle size obtained from the peptide-CpG-ODN complex was around 150 nm, which was an optimal size for the delivery to DCs, which was not the case for free peptide^26^.

It is worth mentioning that the amount of antigen we used for immunizations (2.86 µg/dose) was set to be close to the minimum dose that was needed to trigger T cell proliferation in vitro^19^. On the other hand, in preclinical vaccination studies, higher amounts of CpG-ODN were usually used to elicit a solid immune response^27,28^. In contrast to these studies, we used rather a lower dose of CpG-ODN (800 ng/dose) in both liposomal and non-liposomal formulations. With such a low dose of peptide and CpG-ODN, vaccination with peptide-CpG-ODN complex pulsed DCs substantially controlled the tumor growth progression compared to the free peptide treated group. This demonstrates that in the presence of an anti-PD-1 antibody, CpG-ODN converts DCs into an effective vaccination strategy, generating a potent T cell response able to infiltrate into tumor sites. In addition, we have shown a high amount of IFN-γ production by CD8+ TILs, which is in agreement with other studies which claim the degree of CD3+ CD8+ proliferating TILs in melanoma tumor lesion correlates with the presence of IFN-γ signature, which is associated with intuitive ability in remission of primary lesions^29^.

We also utilized liposomes which are best known for enhancing the activities of various immunoadjuvant compounds, to increase the therapeutic efficacy conferred by CpG-ODN^30^. Several studies have proved that liposomes are an effective delivery vehicle that could improve the CpG-ODN half-life and increase Th1 immune responses^31^. We used a cationic lipid, DOTAP, in the liposomal formulation and then conjugated gp100 peptides to the surface of liposomes as previously described in details^19^. DOTAP was more effective in enhancing CpG-ODN mediated immunity attributed to electrostatic interactions between the positively charged DOTAP and the negatively charged CpG-ODN^32^. Our results showed that incubation of liposomal peptide with CpG-ODN resulted in nanoparticle formation with a size of 226 nm and a negative zeta potential around − 11 mV due to the presence of CpG-ODN. According to our previous studies^27,33^, incubation of liposomes with adjuvants with the appropriate molar ratio was desirable for the liposomal- CpG-ODN stability. It prevents aggregation, and their mixture remained stable that was confirmed by the size and PDI results. In addition, cationic liposomes incubation with the CpG-ODN just before exposure to DC provided an effective presentation of antigen and adjuvant to dendritic cells. The good results of this group are perhaps for these reasons. As shown in our previous study, the in vivo administration of liposomal formulation with CpG-ODN to melanoma tumor challenged mice conferred improved survival compared to non-liposomal plus CpG-ODN treated mice^28^.

Injection of liposomal peptide plus CpG-ODN pulsed DC vaccine combined with anti-PD-1 significantly enhanced infiltration of CD8+ T cells compared to single treated and non-liposomal peptide plus CpG-ODN group, which likely reflects both infiltration and expansion at the tumor site. Our study also demonstrated that TILs from mice received combination therapy with liposomal peptide plus CpG-ODN showed the highest IFN-γ production by CD8+ TILs. Additionally, the liposomal plus CpG-ODN group with anti-PD-1 had a significant increase in the ratio of CD8+/Treg T cells, indicating activated anti-tumor T cell proliferation. We also observed that the B16F10 tumor cells were recognized by antigen-specific T cells and could lead to specific cytotoxicity in this group. Gene expression profiling showed an increased significance of IFN-γ level, suggesting increased T cell migration and IFN-γ production in this group. Our results are in agreement with that of Mansour et al.^34^, indicating that vaccinations with VacciMax (VM), a liposome-based vaccine delivery composed of TRP2, PADRE, and CpG-ODN, markedly stimulates a robust anti-tumor immune response. They also suggested that the presence of CpG-ODN in VM increased the antigen-specific CTL response by approximately twofold.

CD25+ Foxp3+ Treg cells and Myeloid-derived suppressor cells (MDSCs) are major components in mediating the immune-suppressive tumor microenvironment. The high frequency of Tregs is a crucial feature associating with potent inhibition and dysfunction of activated T cells and CD8+ cytotoxic T lymphocytes^35,36^. It was reported by Lai et al*.*^37^ that vaccination strategy by a mannosylated liposomal formulation containing CpG-ODN (M/CpG-ODN-TRP2-Lipo) could significantly reduce the number of regulatory T cells while simultaneously increasing the number of IFN-γ-producing T cells and antigen-specific CD8+ cytotoxic T cells. Based on our findings, a significant decrease in the frequency of CD25+ Foxp3+ Treg cells in the spleen and tumor site denoted that the current combination restricted the pre-existed tumor-supportive immunity with the significantly lower secretion of IL-10 in this group than single treated and non-liposomal peptide plus CpG-ODN groups.

Another critical aspect of IFN-γ production is its positive correlation in combination therapy with monoclonal antibodies. It was reported that a combination of anti-4.1BB antibody with Trp2 peptides and CpG-ODN induced antigen-specific IFN-γ, and CD8+ CTL responses significantly increased up to 75% anti-tumor cure rate, highlighting the importance of TLR9 agonist combination with 4.1BB blockade for achieving tumor eradication^38^. It is, therefore, likely that in our study, liposomal peptide plus CpG-ODN treated mice undergo T cells activation, leading to increased killing of tumor cells, which controlled tumor growth and improved survival rate of treated mice.

## Conclusion

In conclusion, the current study demonstrated that besides the adjuvanticity of CpG-ODN, the enhanced immune response was contributed to the impact of peptide-CpG-ODN complex size and their improved cellular uptake in improving the therapeutic efficacy of anti-PD-1 against melanoma cancer. Our results showed that incubation of liposomal peptide with CpG-ODN combined with anti-PD-1 therapy alleviated immune suppression of tumor microenvironment, enhanced priming of T cells, and increased the efficacy and durability of the treatment. Our results also linked the production of the IFN-γ and enhanced antigen presentation with mechanisms of tackling resistance to PD-1 blockade therapy. Our findings strongly suggest that the liposomal peptide with CpG-ODN formulation develops novel DC-based vaccines as a combinatorial strategy in melanoma cases with resistance to anti-PD-1. We believe that the combination of liposomal peptide plus CpG-ODN pulsed DC vaccine with other existing monoclonal antibodies is worthy of further exploration.

## Material and methods

### Materials

1, 2-distearoyl-Sn-glycero-3-phosphoethanolamine-*N*-[maleimide (polyethylene glycol)-2000] (DSPEPEG2000-Maleimide) and *N*-[1-(2, 3-Dioleoyloxy) propyl]-*N*, *N*, *N*-trimethylammoniummethyl-sulfate (DOTAP) were purchased from Avanti Polar Lipid (Alabaster, AL, USA). Cholesterol, Hyaluronidase enzyme, and Lipopolysaccharides (LPS) were purchased from Sigma-Aldrich (Steinheim, Germany). Recombinant Mouse IL-4 and GM-CSF were purchased from Biolegend (San Diego, USA). Calcein AM (AM = acetoxymethyl) and Phytohemagglutinin (PHA) were purchased from Invitrogen (Carlsbad, CA). Collagenase Type I enzyme was purchased from Gibco (UK). Flow cytometry kits and antibodies were purchased from BD Biosciences (San Diego, USA). All other solvents and reagents were used as a chemical grade.

### Synthetic peptide, CpG-ODN, and monoclonal antibody

The gp100 _25–33_ CD8 T cells epitope, containing linker sequence (AcCGGGEGPRNQDWL) with 95% purity was synthesized and identified with mass spectrometry analysis and high-performance liquid chromatography (China Peptides Co, Shanghai, China). Synthetic ODN-1826 containing CpG motifs (5′-tccatgacgttcctgacgtt-3′) with a nuclease-resistant phosphorothioated backbone was obtained from Bioneer Co (Korea). The anti-PD-1 antibody generated from the J43 clone was obtained from BioXCell Co (West Lebanon, USA).

### Cell lines and mice

The B16F10 melanoma cell line and CT26, a murine colon carcinoma cell line (gp100 negative), were obtained from Pasture Institute (Tehran, Iran) and maintained in DMEM and RPMI cell culture containing 10% fetal bovine serum (FBS) and antibiotics.

C57BL/6 female mice at the age of 6–8 weeks were purchased from Royan Institute (Tehran, Iran). All mice were maintained under pathogen-free conditions with water, and food was given ad libitum.The animal protocols was approved by Mashhad University of Medical Sciences in accordance with the Ethical Committee and Research Advisory Committee (Grant number: 941426).This study was carried out in compliance with the ARRIVE guidelines.

### DC-based vaccine preparation

Cationic liposomes (DOTAP: Chol) were prepared by thin lipid film hydration method, and then the gp100 peptide was conjugated to them as was utterly explained in our previous study^19^. For those treatment groups containing CpG-ODN, CpG-ODN was mixed with liposomal or non-liposomal gp100 and left for 30 min at room temperature before taken up by DCs (ratio of CpG-ODN per total lipid: 0.002 µmol per 0.24 µmol)^27^. The constant concentration of CpG-ODN was used for CpG-ODN formulations (Table S1). The final CpG formulations in the presence of liposomal or non-liposomal gp100 were analyzed for particle size, zeta potential, and polydispersity index (PDI) using the dynamic light scattering (DLS) instrument (Malvern, UK).

Murine BM progenitor cells were used for DCs preparation according to the previously described method^19,39^ and characterized for maturation state. 5 × 10^5^ matured DCs (by LPS) (Fig. S4) were incubated with either formulation for 1 h, and then primed DCs were washed and resuspended in PBS before administration. The peptide concentration was maintained constant, whether in non-liposomal or liposomal form (Table S1).

### Mouse model study

For evaluation of in vivo efficacy of PD-1 blockade in combination with DC vaccine, mice were challenged with 3 × 10^5^ live B16F10 melanoma cells via subcutaneous (s.c.) route on the right flank. About ten days after tumor challenge (tumor size ~ 0.5 cm), mice were randomly assigned into five groups (10 mice). Tumor-bearing mice were given three doses of vaccines (priming dose followed by two other booster doses on days 10 and 17 and 24, respectively) s.c. in the groin. Mice were also given four intraperitoneal (i.p.) injections (on days 11, 15, 19, 23) of PD-1 blockade, 100 µg per dose. All the injections were adjusted to 100 µl volume in PBS. DC pulsed with PBS was used as a control group.

Mice were observed every two days for tumor growth and mortality for the next 25 days. Tumor growth was monitored using a set of calipers for measuring their perpendicular diameters. Tumor volume was calculated as length × width × height × Л/6. Tumor-bearing mice that died on their own or became ulcerated or surpassed 1000 mm^3^ volume were excluded. For survival experiments, mice with a tumor volume of more than 1000 mm^3^ were considered moribund. For each treated group, TTE, TGD, MST, and ILS were calculated^40,41^. The experiment was terminated on day 26, and three mice from each treated group were used for immunological analysis.

### Tissue harvest and single cell preparation

Spleens and tumors were dissected from mice on day 26, 48 h after the third DC vaccination. Tumors were digested in Collagenase type I and Hyaluronidase for 2 h at 37 °C. Spleens and enzymatically digested tumors were mashed through a 70-µm cell strainer on ice-cold PBS and centrifuged at 1600 × g for 5 min. ACK lysing buffer used for lysing RBC, and the single cells were used for immunological analysis.

### Intracellular staining and FACS assay

Spleen and single tumor cells were stimulated with ten µg of gp100 peptide containing epitope for major histocompatibility complex (MHC) class I for 24 h, also the addition of brefeldin A (6 h), and then washed and divided into different tubes (2 × 10^5^) and stained for CD8+ T cells, helper T cells (Th) and regulatory T cells (Treg) according to the manufactures structure. Cells were analyzed for the IFN-γ CD8 or CD4 positive, IL-4 CD4 positive T cells, and IL-10 Foxp3 positive Tregs on a flow cytometer (BD). All experimental determinations were performed in triplicate.

### Antigen-specific cytotoxic assay

Spleen single-cell suspensions (Effector cells) were co-cultured with Calcein AM (12.5 µM) labeled B16F10 (gp100 positive) and CT26 (gp100 negative) cell lines (Target cells, 2 × 10^4^ cells per well) to evaluate the cytotoxic activity CD8+ T cells. Mixed cell suspensions at different E:T ratios were plated in 96-well plates (triplicate) in complete DMEM. At four h of culture at 37 °C, cells were centrifuged, and the fluorescence supernatants were analyzed with excitation at 485 nm and emission at 538 nm using a fluorescent plate reader (FLx800, BioTek Instruments Inc. USA). The percentage of lysis was calculated as was mentioned previously^42^.

### T-cell assay via ELISpot

The T cells' response to treatment was also determined by the secretion of cytokines followed by antigen stimulation. The numbers of T cells producing IFN-γ and IL-10 were determined by Enzyme-Linked Immunospot Assay. For these experiments, 3 × 10^5^ spleen or tumor cells were stimulated with 10 µg/ml peptide in cell culture for 24 and 48 h, respectively. 10^5^ cells stimulated with PHA (10 µg/ml) were considered as positive control, and cell without any stimulation were considered as a negative control. The cytokine spot-forming units (SFU) were visualized according to the manufacturer’s protocol of mouse IFN-γ and IL-10 basic ELISpot kits (Mabtech, Sweden) and counted using Kodak 1D image analysis software (Version 3.5, Eastman Kodak, Rochester, New York, USA). The normalized results were expressed as spots per 10^6^ cells.

### Gene expression level assay using real-time PCR

Total RNA in spleens and tumors were isolated via the Column RNA isolation kit (Denazist, Iran). For assessing the expression level of IFN-γ and IL-10, cDNA was reverse-transcribed from mRNA using cDNA synthesis kit (Yekta Tajhiz Azma, Iran). cDNA samples were amplified in triplicate in a Rotor gene Q instrument (QIAGEN Hilden, Germany) using the two step Sybr Green real-time PCR kit (Yekta Tajhiz Azma, Iran). The primer sequences used are as follows: IFN-γ Forward: 5′GCTCTGAGACAATGAACGCT3′, Reverse: 5′AAAGAGATAATCTGGCTCTGC3′; IL10 Forward: 5′TGAGAACAGCTGCACCCACT3′, Reverse: 5′GGAAACCCAGGTAACCCTTA3′; and GAPDH Forward: 5′TGCACCACCAACTGCTTAG3′, Reverse: 5′GATGCAGGGATGATGTTC3′. Fold changes of mRNA expression level among groups were determined and normalized using GAPDH expression as the reference. All the procedures were done according to the manufacturer’s guidelines of kits.

### Immunohistochemistry (IHC) assay

Tumor tissues was fixed in formalin for 24 h before being embedded in paraffin wax. Formalin-fixed paraffin embedded (FFPE) 5-μm murine derived tumor tissue sections was used for Immunofluorescent staining as described previously^19^ using PD-1 antibody (1/400, 15 min in dark). For visualizing primary antibody, the Mouse/Rabbit PolyVue Plus™ HRP/DAB Detection System kit (Diagnostic Bio Systems, Pleasanton, CA, USA) was used, and nuclei were visualized with Hematoxylin.

### Statistical analysis

Data were analyzed using a two or one-way ANOVA for multiple comparisons with Tukey as post hoc test. All analyses were performed using GraphPad Prism Software version 8. Statistical analyses for evaluating the survival advantage were performed using log-rank tests. Differences were considered statistically significant if *P* < 0.05.

## Supplementary Information

Supplementary Informations.

## Data Availability

The data that support the findings of this study will be made available upon reasonable request.
