# Supplementary material for: Ex vivo dendritic cell-based (DC) vaccine pulsed with a low dose of liposomal antigen and CpG-ODN improved PD-1 blockade immunotherapy
Source: Sci Rep. 2021 Jul 19;11:14661. doi: 10.1038/s41598-021-94250-0 (PMC8290007; doi:10.1038/s41598-021-94250-0)
Supplement: Supplementary file 1 — Supplementary Informations. [file 41598_2021_94250_MOESM1_ESM.docx]

***Ex viv*o dendritic cell-based (DC) vaccine pulsed with a low dose of liposomal antigen and CpG-ODN improved PD-1 blockade immunotherapy**

Mona Yazdani a, Zahra Gholizadeh b, Amin Reza Nikpoor c,d , Nema Mohamadian Roshan e, Mahmoud Reza Jaafari f,g, Ali Badiee a,g *

a Nanotechnology Research Center, Pharmaceutical Technology Institute, Mashhad University of Medical Sciences, Mashhad, Iran

^b^ Department of Molecular and Comparative Pathobiology, Johns Hopkins University School of Medicine, Baltimore, MD 21205, USA

^c^ Molecular Medicine Research Center, Hormozgan Health Institute, Hormozgan University of Medical Sciences, Bandar Abbas, Iran

^d^ Immunogenetic and Cell Culture Department, Immunology Research Center, School of Medicine,

Mashhad University of Medical Sciences, Mashhad, Iran

^e^ Department of Pathology, School of Medicine, Mashhad University of Medical Sciences, Mashhad, Iran

^f^ Biotechnology Research Center, Pharmaceutical Technology Institute, Mashhad University of Medical Sciences, Mashhad, Iran

^g^ Department of Pharmaceutical Nanotechnology, School of Pharmacy, Mashhad University of Medical Sciences, Mashhad, Iran

* **Corresponding author:**

Ali Badiee (Pharm. D., Ph. D.)

Associate Professor in Pharmaceutics,

School of Pharmacy, Mashhad University of Medical Sciences, Mashhad, Iran

P.O. Box: 91775-1365

Phone: 0098-513-1801339 (Office)


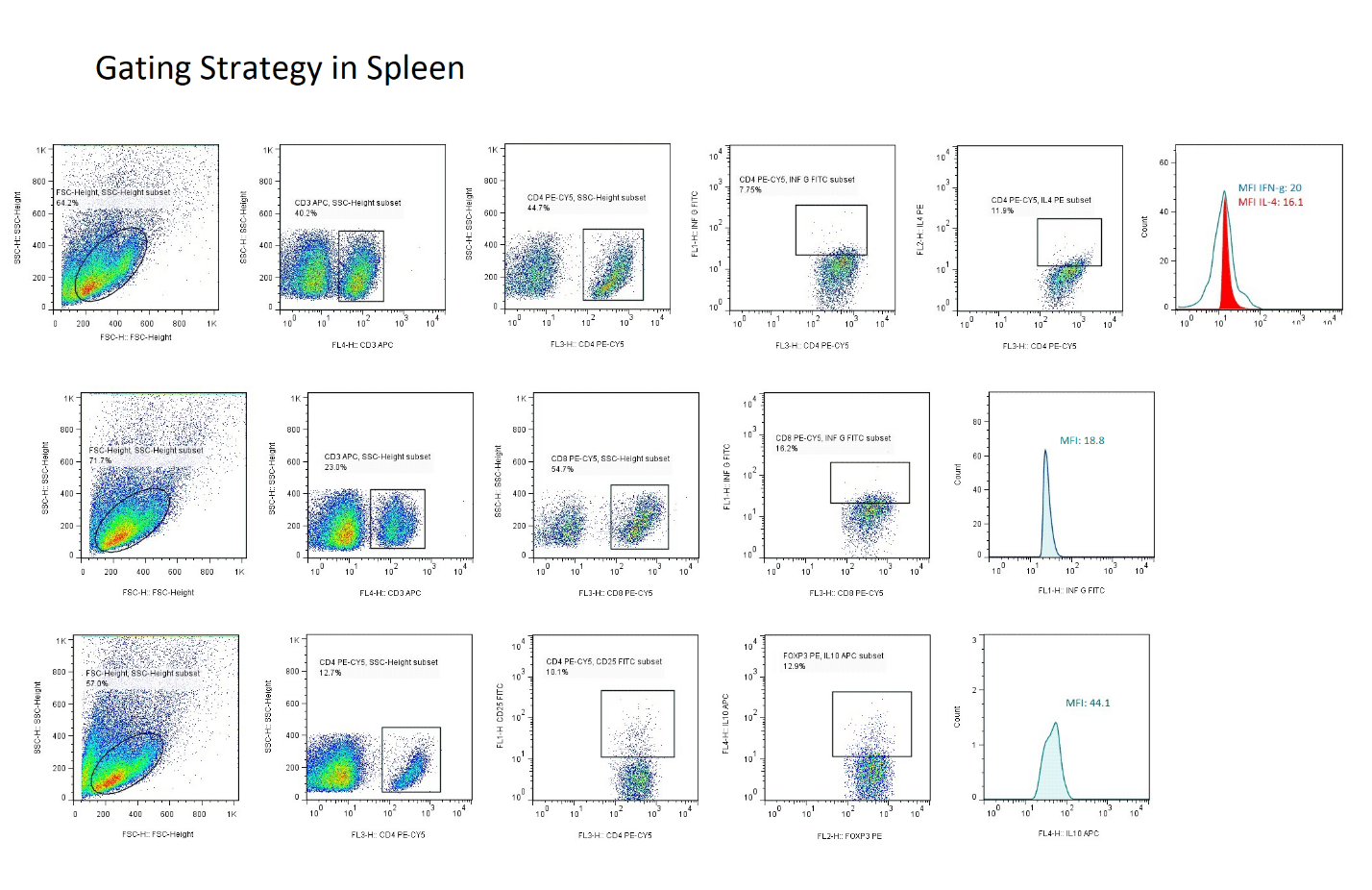


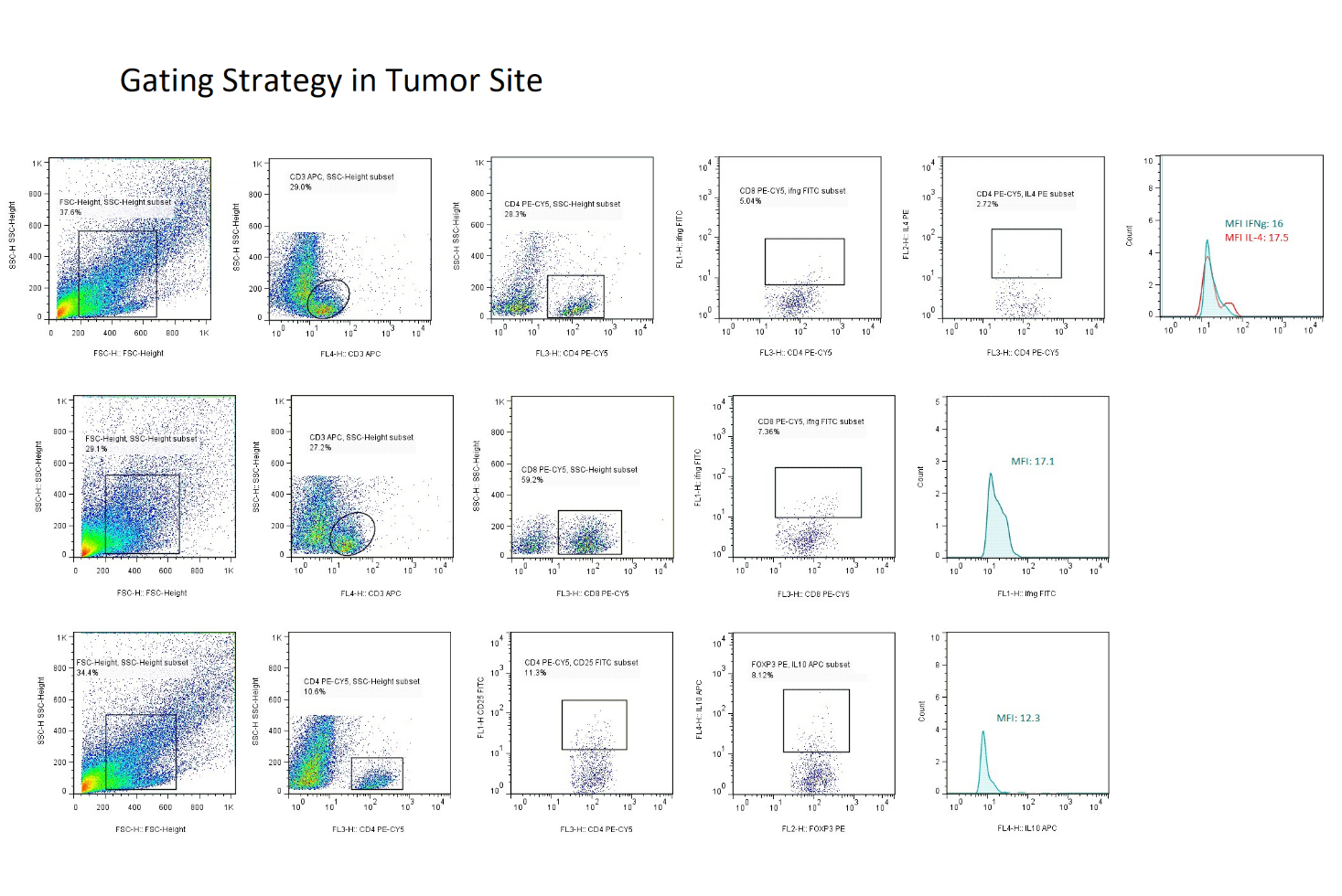


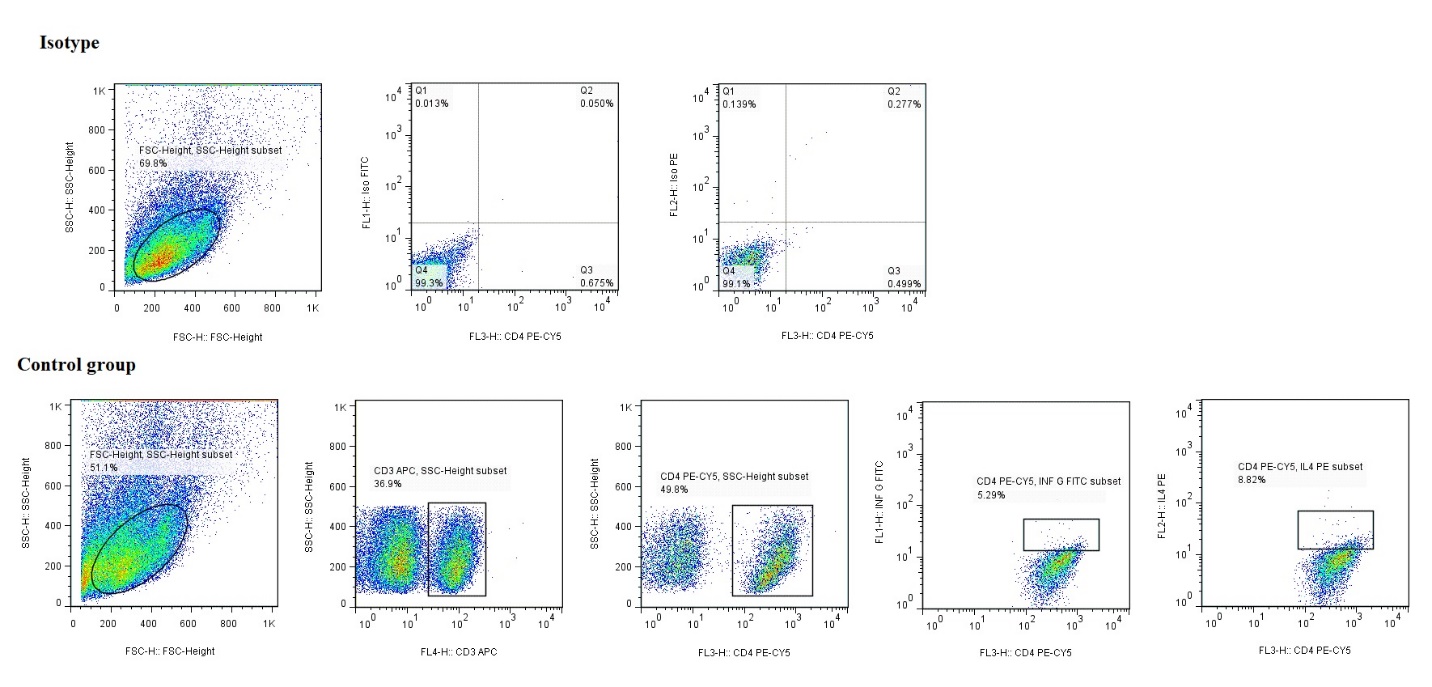


**Figure S1: Representative dot plots of CD8, CD4 and Tregs in** **different groups at day 25.** Spleen and tumor tissues obtained from different groups were stained with fluorochrome-labeled antibodies against CD3, CD4, CD8, CD25 and FoxP3. For CD8+ and CD+ T cells, acquired cells were first gated on CD3+ T cells followed by gating for CD4+ or CD8+ cells within CD3+ T cells for next analysis, percentage of cytokine producing cells and mean fluorescence intensity. The cells expressed CD25+ FoxP3+ within CD4+ T cells was determined as Tregs. Representative dot plot of Isotype and control group (DC+Buffer).


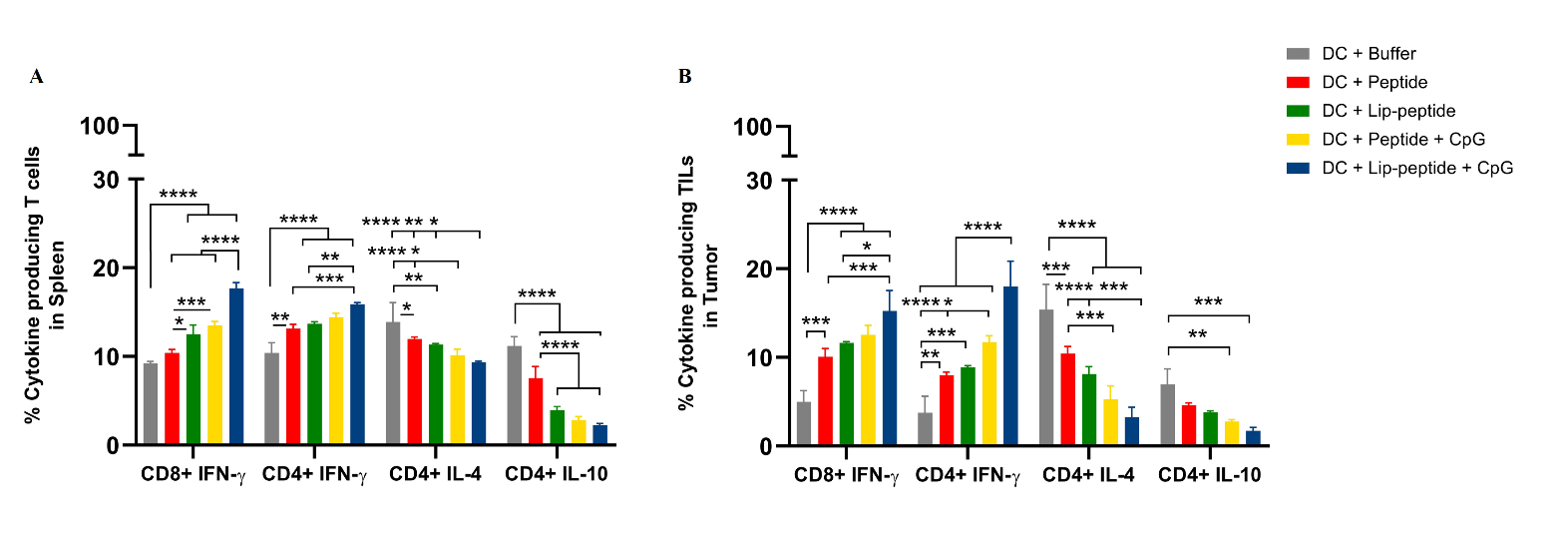


**Figure S2: The percentage of cytokine producing cells in spleen and tumor site.** The percentage of splenic CD8 gated T cells expressing IFN-γ, CD4 gated T cells expressing IFN-γ, IL-4 and IL-10 (A). The percentage of CD8+ expressing IFN-γ and CD4+ expressing IFN-γ, IL-4 and IL-10 in expanded TILs (B).


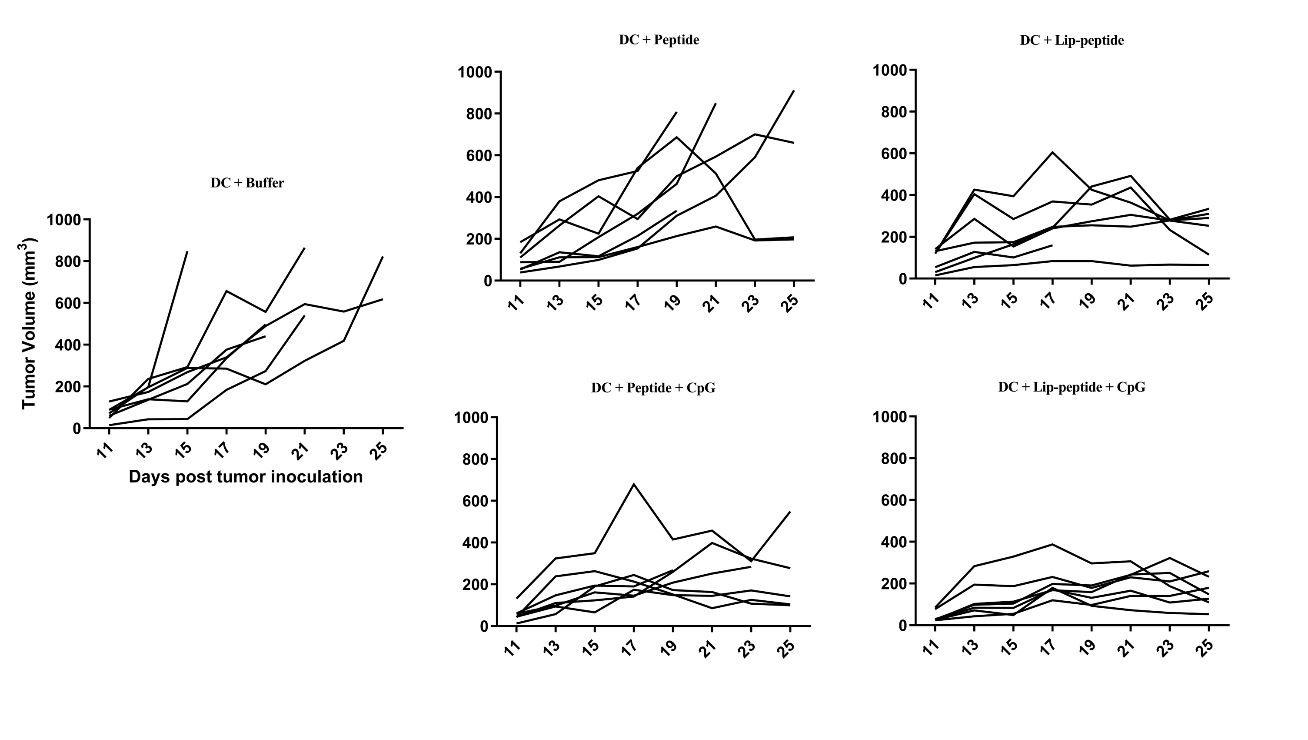


**Figure S3: Tumor volume of each mouse in different treated groups during 25 days follow up.**


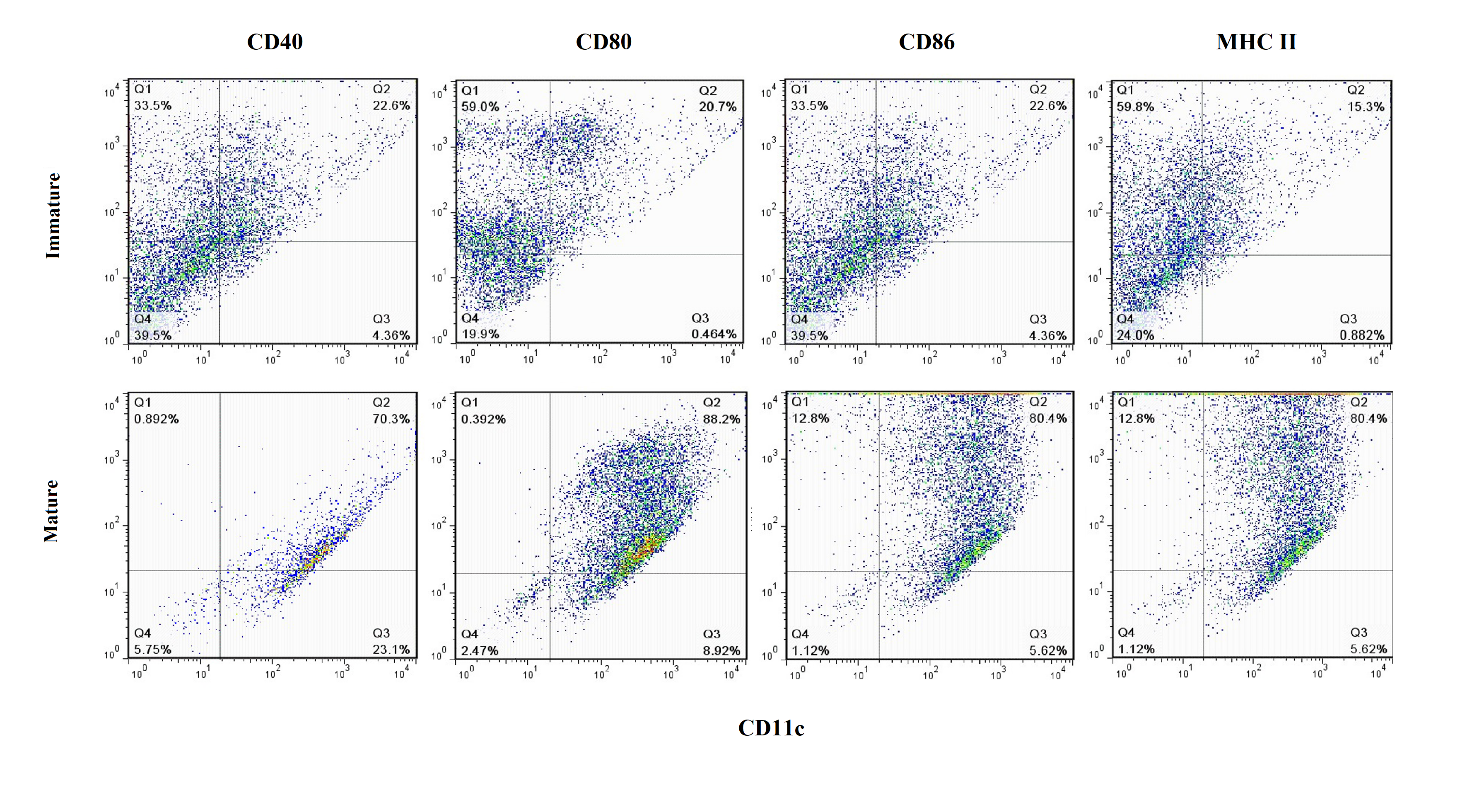


**Figure S4: Flow cytometric analysis of dendritic cells CD markers on day 5 as immature and day 7 as mature DCs.**

**Table S1: The dose of DOTAP lipid, GP100 peptide and CpG-ODN adjuvant in different administered formulations.**

| Groups | DOTAP dose (nmol/mouse) | GP100 dose (µg/mouse) | CpG-ODN dose  (µg/mouse) |
| --- | --- | --- | --- |
| Peptide | **-** | 2.8 µg | **-** |
| Peptide + CpG-OGN | **-** | 2.8 µg | 0.8 µg |
| Lip-peptide | 15 nmol | 2.8 µg | **-** |
| Lip-peptide + CpG-ODN | 15 nmol | 2.8 µg | 0.8 µg |
